# Initial clinical evidence on biperiden as antiepileptogenic after traumatic brain injury—a randomized clinical trial

**DOI:** 10.3389/fneur.2024.1443982

**Published:** 2024-08-07

**Authors:** Maira Licia Foresti, Eliana Garzon, Mariana Teichner de Moraes, Rafael P. S. Valeriano, João Paulo Santiago, Gustavo Mercenas dos Santos, Natália Mata Longo, Carla Baise, Joaquina C. Q. F. Andrade, Maria Alice Susemihl, Claudia da Costa Leite, Maria da Graça Naffah Mazzacoratti, Wellingson Silva Paiva, Almir Ferreira de Andrade, Manuel Jacobsen Teixeira, Luiz E. Mello

**Affiliations:** ^1^Neurology Neuroscience Postgraduation Program, Physiology Department, Escola Paulista de Medicina, Universidade Federal de São Paulo, São Paulo, Brazil; ^2^Instituto D’Or de Pesquisa e Ensino, São Paulo, Brazil; ^3^Department of Neurology, Hospital das Clínicas, Faculdade de Medicina, Universidade de São Paulo, São Paulo, Brazil; ^4^Sociedade Beneficente de Senhoras Hospital Sírio-Libanês, São Paulo, Brazil; ^5^Associação Brasileira de Epilepsia, São Paulo, Brazil

**Keywords:** acute traumatic brain injury, post traumatic epilepsy, seizure, epilepsy, anticholinergic, biperiden

## Abstract

There is currently no efficacious intervention for preventing post-traumatic epilepsy (PTE). Preclinical studies support the potential use of anticholinergics for this condition. The purpose of this study was to evaluate the effects of biperiden as an intervention for preventing PTE. A randomized, double-blinded clinical trial was conducted at HC/FMUSP between 2018–2022. Adults with acute traumatic brain injury (TBI) were randomly assigned to receive biperiden or placebo, for 10 days. The primary outcome was the incidence of PTE while the secondary outcomes included the frequency of seizures, the frequency of any adverse events and mortality after 24 months. The study was powered at a planned enrolment of 132 patients. The trial began in January 2018 and was halted by researchers on March 2020 (and terminated in December 2022) in the face of the global COVID-19 pandemic. Overall, 123 participants were randomized and 112 contributed with data for modified mITT analysis, being that 61 (49.5%) participants completed the 24-month follow-up consult. Data analysis indicated lack of evidence of biperiden for either, the incidence of post-traumatic epilepsy (2.6, 95%CI, 0.65–10.57; *p* = 0.170) or the mortality rate (1.57, 95%CI, 0.73–3.38; *p* = 0.248). The frequency of late post-traumatic seizures was higher for biperiden group (2.03, 95%CI = 0.912–3.1597; *p* <0.001). The present study suggests that there was insufficient evidence regarding the effect of biperiden in preventing PTE after TBI, which underpins the need for larger studies.

**Clinical trial registration: **ClinicalTrials.gov, identifier: NCT01048138.

## Graphical abstract

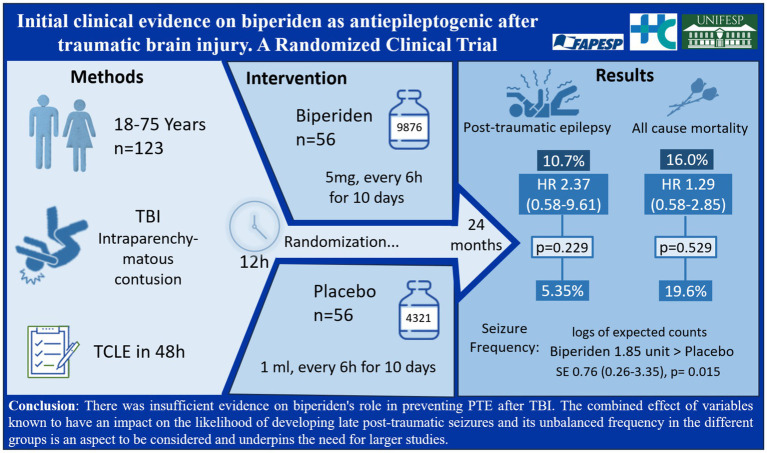

## Introduction

Traumatic brain injury (TBI) has about 27 million new cases globally, resulting in 346 cases per 100,000 population incidence rate, according to the most up-to-date systematic analysis for the Global Burden of Disease (GBD) of TBI (1). One of the major neurological sequelae associated with TBI is post-traumatic epilepsy (PTE), which is characterized by recurrent, unprovoked seizures, starting within months to years after TBI (2). The incidence of PTE has been difficult to define owing to inadequacies in seizure reporting, long-term follow-up and variations in study approaches (2), but it is estimated that affects 2.1, 4.2, 16.7 to 50% of patients with mild, moderate, severe and penetrating TBI (3, 4). PTE impairs neurological recovery after TBI and is independently associated with poor functional outcomes (5).

Despite the available knowledge, there is currently no effective and safe intervention to prevent PTE, and no clinically available medications that have direct actions on the underlying disease process leading to seizures or its progression (6). Indeed, recent terminology recommendations proposed by the International League Against Epilepsy indicate that the pharmacological class that was previously known as antiepileptic drugs, should be named as antiseizure medications (ASM). This change recognizes the fact that ASM are unable to suppress the underlying medical condition, that is epilepsy (7). In fact, only a few antiseizure medications have ever been evaluated for the prevention of PTE in randomized controlled trials (RCTs), and all failed (8). Still, agents that are approved for use in a number of conditions in humans have been successful in affecting disease progression in animal models of epilepsy but have yet to be tested in the clinical setting as antiepileptogenic agents (9). In fact, although the safety profile of repurposed drugs is much better understood than for a new molecule, the availability of studies assessing the second use of an already existing medication, is less than it would be desired.

Original research revealed the potential antiepileptogenic effects of anticholinergic drugs (10). In these studies, drugs that modify neuronal plastic processes, such as anticholinergic agents (e.g., antimuscarinic compounds), have shown the potential to modify the natural course of post-traumatic epilepsy, by decreasing the incidence and intensity of spontaneous epileptic seizures and delaying their appearance in animal models of epilepsy (10). Here, we provided a first assessment of the use of an antimuscarinic compound into real-world use, by investigating whether biperiden, a widely used drug for other pathologies (e.g., Parkinson), in patients after TBI.

In addition to being a routine antiparkinsonian agent with decades of use in millions of patients worldwide (11), there have also been reports on the experimental use of biperiden for the treatment of depression (12, 13). The available evidence indicates its effect on Parkinson’s to be associated with modulation of the cholinergic neurons in the striatum (14). In contrast, its potential use as an antidepressant has yet to be understood, despite some suggestions of an effect via BDNF/TrkB signaling (15). For its purported application as an antiepileptogenic or disease-modifying agent, the testing hypothesis revolves around the modulation of plastic phenomena (10).

Therefore, considering that TBI leading to PTE provides the best opportunity for investigating epileptogenesis employing a parallel animal/human research paradigm (16), the primary goal of this clinical study was to present preliminary outcomes from a double-blind, randomized, placebo-controlled study. This study aimed to evaluate the effects, both beneficial and adverse, of administering biperiden to individuals with TBI as a preventive measure against PTE.

## Methods

### Design and setting

This was a randomized, double-blinded clinical trial, conducted at Hospital das Clínicas da Faculdade de Medicina, Universidade de São Paulo (HC-FMUSP) in collaboration with Hospital São Paulo, Universidade Federal de São Paulo, Brazil, between 2018 (31/01/2018) and 2022 (21/12/2022). The study protocol was registered with ClinicalTrials.gov (NCT01048138) available from: https://www.clinicaltrials.gov/study/NCT01048138.

This report followed the recommendation of the Consolidated Standards of Reporting Trials (CONSORT) checklist (17).

### Ethical aspects

The study protocol was approved by the Local Research Ethics Committee at HC-FMUSP (number 08533513.6.2002.0068) and Unifesp (number 08533513.6.1001.5505). All patients enrolled in the study provided informed consent. Alternatively, inclusion in the study was granted by a legal representative.

### Participants

Patients with acute TBI admitted at the emergency care unit (ECU) of the HC-FMUSP, between January 2018 and December 2020 were screened. Screening procedures, including standard computerized tomography (CT) scan evaluation, were performed by the resident neurosurgeon involved in the care of the patient to determine subject eligibility criteria.

Inclusion criteria were age 18–75 years; diagnosis of acute TBI admitted to the emergency unit within 12 h of the trauma, regardless of the accident; brain CT scan with signs of acute intraparenchymatous contusion; signed informed consent (possibly by a relative, within 48 h after inclusion). Exclusion criteria were history of epilepsy or use of anti-seizure medication (ASM); previous history of TBI; previous cerebrovascular accident; malignant neoplasia and other severe comorbidities; neurodegenerative disorders; concomitant use of other anticholinergic medications; pregnancy; presence of any factor that might contraindicate the use of biperiden; current inclusion in another clinical trial. Alcohol intoxication did not lead to exclusion of the subject.

### Sample size

In order to detect a reduction in the incidence of PTE from 23% in the placebo group to 5% in the biperiden group, with an alpha risk of 5% and power of 80%, this trial was originally designed to enroll 132 patients, being 57 patients in each treatment arm (placebo and biperiden) and an additional 18 patients to compensate eventual screening failure or follow-up loss. However, recruitment and funding issues, and mainly the SARS-CoV-2 pandemic prompted an adjustment in the study design to stop enrollment at 123 patients.

### Randomization, allocation concealment, and blinding

Randomization was performed using random numbers generated by the SPSS statistical package (14.0 for Windows, SPSS Inc), and was done at a 1:1 ratio of placebo and biperiden, in blocks of 6, by an unblinded investigator. Each ampoule was tagged according to the randomly allocated number. Next, tagged ampoules were organized in crescent order and sequentially dispensed by a blinded pharmacist, following the sequential inclusion of participants admitted in the ECU.

Biperiden was presented at an amber ampoule of 1 mL (5 mg/mL; Cinetol, Cristália, Brazil) and had its commercial tag replaced by a numbered tag (following random generated numbers, as already described). Placebo consisted of 1 mL of sterile 0.9% saline solution. Although saline solution had similar color, odor and texture of biperiden, placebo amber ampoule was slightly larger (11.9%) than biperideno ampoules owing to specificities of the production machinery. Except for this, both color and shape of the placebo ampoule, as well as the numbered tag, were identical to the biperiden ampoule. Therefore, care providers (managing physicians and nurses) were partially blinded, given that despite lacking awareness of group assignment, those minimal bottle size differences could be recognized if closely compared. Study medication was stored and dispensed by the hospital pharmacy service to the nurses engaged in the trial, which also kept the records of the distribution of medications used in this clinical trial, to provide drug accountability.

### Intervention

Once the patient was enrolled, 1 mL of the study randomized medication, biperiden or placebo, was diluted in 10–50 mL of sterile saline, and intravenous administered by assistant nurses as soon as possible within 12 h after TBI, aiming at modifying the epileptogenic process. The intervention was repeated every 6 h for 10 consecutive days, until completing 40 total doses (10, 18).

Demographic and clinical data of the patients were collected, including the occurrence of acute symptomatic seizures (seizures occurring immediately after TBI until 7 days after trauma). The incidence of already known clinical adverse events for biperiden use and other events were evaluated during the intervention period. Patients were not deprived of any medical treatment, including use of ASM (such as phenytoin, mainly during the acute hospitalization period when presenting with acute symptomatic seizures following TBI), indicated for their case.

For the follow up, clinical evaluation was performed by blinded experienced epileptologists at 1, 3, 6, 12, 18 and 24 months after TBI. At each visit, any adverse events were assessed, and neurologic examination was performed, focusing on the occurrence of unprovoked epileptic seizures, including absence seizures, starting 7 days after TBI, which characterize PTE. Therefore, the PTE diagnosis was defined based on detailed clinical history that was obtained from the patient and family members, guided by experienced epileptologists regarding key points for seizure diagnosis.

It is important to highlight that, unfortunately, due to the restrictive social measurements imposed by the SARS-CoV-2 pandemic in 2020/2021, the in person follow up assessments to verify history of seizure occurrence, and its clinical characteristics in order to classify seizures types, had to be replaced by phone calls, which continued to be performed by the same epileptologists.

### Outcomes

Primary outcome was the incidence of PTE. The incidence of PTE was evaluated by identifying spontaneous seizures initiated 7 days after TBI and during the two-year follow-up period. PTE incidence was compared between placebo and biperiden-treated patients at 24 months.

Secondary outcomes: i. Frequency of seizures. The frequency of seizures was counted starting 7 days after TBI and during the two-year follow-up period. Frequency of seizures was compared between placebo and biperiden-treated patients at 24 months after TBI. ii. Mortality and adverse effects. The incidence of death and adverse effects was counted starting immediately after TBI and continuously, during the intervention period (for the adverse events), or the two-year follow-up period (for mortality). Incidence of death and adverse effects was compared between placebo and biperiden-treated patients at 10 days and 24 months after TBI, respectively.

As part of an exploratory extended study, which results will be reported separately, acute and chronic electroencephalogram (EEG), genetic and behavioral data were also monitored for assessing potential mechanisms by which biperiden might exert its actions on epileptogenesis.

### Statistical analysis

For continuous outcomes, means and standard deviation (SD) values are presented. For categorical data, proportions and counts are presented. For an even more precise characterization of study participants, it is presented the features of age and Glasgow Coma Scale on scene (GCSoS) of the accident, as well as on hospital admission (GCSoA), both as continuous data, used at statistical analysis, and as categorized data, which is relevant for clinic interpretation.

Cox regression (Breslow method for ties) was used to assess the difference between groups on the two time-to-event outcomes (PTE and mortality). The assumption of proportional hazards was tested via Schoenfeld residuals. Moreover, given that mortality is a competing risk of PTE incidence, we used the competing-risks regression based on Fine and Gray’s proportional subhazards model for the PTE modeling as a sensitivity analysis.

Zero-inflated Poisson regression with robust standard error (19) was used to estimate the effect of the intervention on the seizure frequency (number of post-traumatic seizures), which is the secondary outcome.

The GCSoS was used as an adjustment given that it is described in the literature that GCS can be a predictor of PTE (5, 20) and, therefore, based on a clinical perspective. However, note that both unadjusted and adjusted estimates are reported to give transparency regarding the effects, especially given that the outcomes are time-to-event (21). Due to missing data in GCSoS and to avoid reduction in the power due to missing in covariate GCSoS when running adjusted models, it was used multiple imputations. The following measures were considered in the unrestricted models for the imputation: age, sex, and group randomization. Because all those measures had non-missing values, they were used only as predictors in the unrestricted model. It was imputed ten datasets and the results presented are the pooled estimates. STATA version 14 was used for all the analyses. The statistical significance level adopted was 0.05. Final analyses were performed by a blinded statistician.

## Results

**Figure 1:**
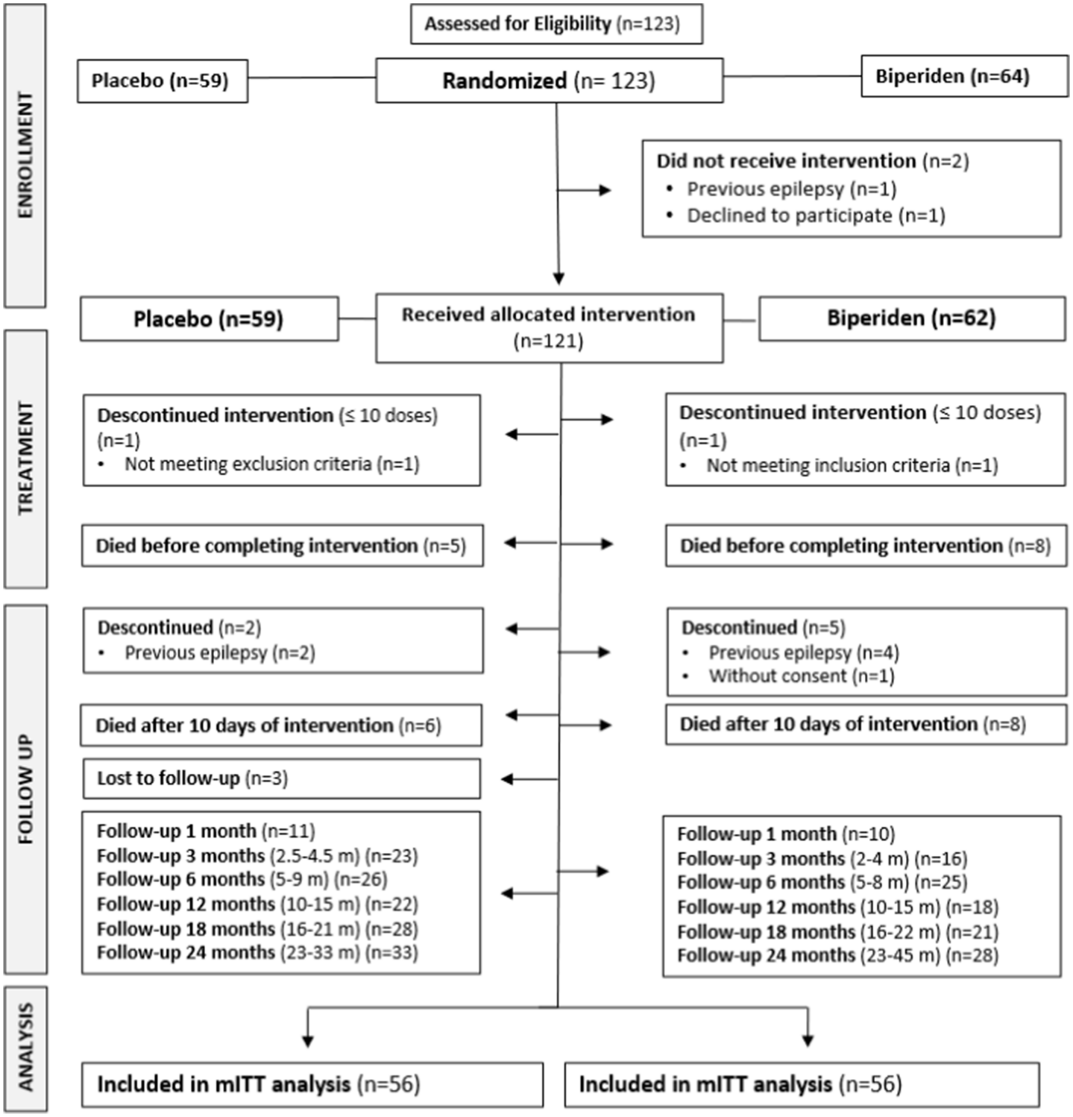
Flow diagram of randomized clinical trial for biperiden as antiepileptogenic after traumatic brain injury.

A total of 122 adult consecutive patients with TBI meeting the inclusion criteria were recruited from January 2018 to March 2020, when patient’s recruitment was suspended due to the social isolation measures imposed by the SARS-CoV-2 pandemic. A single patient was recruited in December 2021, totalizing 123 recruited patients. Of these, 59/123 patients (47.9%) were randomized for the Placebo group and 64/123 patients (52%) were randomized for the Biperiden group (Figure 1). Imbalanced sample sizes can be partially explained by the premature termination of the study. Additionally, we encountered two cases of broken boxes that were discarded, and we continued with the expected sequence of allocation. As already pointed out, with the social restrictions measures implemented as a result of the SARS-CoV-2 pandemic, the monitoring of patients after hospital discharge was interrupted as of March 2020. After a period of reorganization, some clinical follow-ups were carried out through telephone calls, in an attempt to maintain the link between patients and the project team and to monitor the emergence of PTE. Despite being effective, there was a delay in the evaluation of many patients, especially for those completing the follow-up period in 2020/2021 pandemic. A total of 3/123 patients (2.4%) were lost in the follow-up, without completing any visits. A total of 27/123 patients (21.9%) died during the two-year follow-up (placebo *n* = 11, biperiden *n* = 16). In addition, 11/123 (8.9%) patients were excluded or discontinued from the study, mostly for presenting previous epilepsy (*n* = 7), declining participation (*n* = 2) or receiving only a few doses for failing eligibility criteria (*n* = 2), and therefore were not included in the modified mITT analysis. Overall, of the 82/123 (66.6%) remaining participants, a total of 61/123 (49.5%) participants (placebo *n* = 33, biperiden *n* = 28) completed the last study assessment, comprising the 24 months period after TCE (last appointment ranged between 23 and 45 months). Figure 1 shows the flowchart of the trial.

Considering all participants, 22/123 (17.8%) were female with mean (SD) age of 51.7 (20.0) years and 101/123 (82.1%) participants were male, with 42.0 (16.7) years, resulting in 43.7 (17.6) mean (SD) age for the whole sample.

Tables 1, 2 are descriptive statistics for all participants in terms of continuous and categorical measures, respectively. It might be observed that the groups are balanced in terms of demographic and clinical features, including those related to the incidence of ASS. However, it is important to highlight that it was observed some unevenness among groups when considering some clinic aspects relevant for the PTE development, e.g., given the known GCSoS (data available for 85/123 (69.1%) participants), the biperiden group had 35/45 (77.7%) participants with moderate and severe TBI, while the placebo group presented 23/40 (57.5%) participants with this TBI severity. Such distributions are more even if considering the GCSoA, but this must be considered carefully as patients could be under sedation upon arrival at the hospital. Furthermore, contributing to discrepancies among features relevant for PTE development, the number of participants with bilateral brain lesions in the biperiden group (25/64; 39.0%), was double the number of participants in the placebo group (10/59; 16.9%).

**Table 1:** Summary statistics of the baseline characteristics of participants by allocated group.

Characteristic	Control (*n* = 59)		Biperiden (*n* = 64)	
	*n*	mean (SD)	*n*	Mean (SD)
Age, y	59	43.70 (17.68)	64	43.78 (17.66)
GCSoS, score	40	8.75 (4.35)	45	8.51 (4.35)
GCSoA, score	59	7.51 (4.80)	64	7.49 (4.83)
Hospital stay, d	49	29.10 (33.64)	48	28.88 (33.85)

GCSoS, Glasgow Coma Scale on scene; GCSoA, Glasgow Coma Scale on admission.

**Table 2:** Summary statistics of the baseline characteristics of participants by allocated group.

Characteristic		Control (*n* = 59)		Biperiden (*n* = 64)	
		*n*	%	*n*	%
Female		11	18.6	11	17.1
Age	16–45	33	55.9	38	59.3
	46–88	26	44.0	26	40.6
GCSoS	Mild	17	28.8	10	15.6
	Moderate	3	5.08	5	7.8
	Severe	20	33.8	30	46.8
	Missing	19	32.2	19	29.6
GCSoA	Mild	19	32.2	15	23.4
	Moderate	9	15.2	4	6.25
	Severe	31	52.5	45	70.3
Acute symptomatic seizure (yes)		10	16.9	14	21.8
Neurosurgery (yes)		29	49.1	42	65.6
Phenytoin (yes)		37	62.7	48	75.0
Antibiotics (yes)		39	66.1	49	76.5
Family history of epilepsy	Yes	9	15.2	4	6.25
	No	27	45.7	28	43.7
	Not sure	1	1.69	0	0.0
	Missing	22	37.2	32	50.0
*CT evaluation*
	No lesion	7	11.8	8	12.5
	One lesion	27	45.7	16	25.0
	Two lesions	8	13.5	16	25.0
	Multiple lesions	15	25.4	19	29.6
Missing**	2	3.38	4	6.25
Lateralization of lesions*	No lesion	7	11.8	8	12.5
	Right hemisphere	21	35.5	14	21.8
	Left hemisphere	19	32.2	13	20.3
	Bilateral	10	16.9	25	39.0
	Missing**	2	3.38	4	6.25
Location of lesions*	No lesion	7	11.8	8	12.5
	Frontal	30	50.8	35	54.6
	Temporal	26	44.0	34	53.1
	Parietal	7	11.8	6	9.37
	Occiptal	3	5.08	2	3.12
	Nucleocapsular region	1	1.69	0	0.0
	Other (insula, cerebellum)	1	1.69	1	1.56
	Missing**	2	3.38	4	6.25
Fracture	Yes	31	52.5	37	57.8
	No	26	44.0	23	35.9
	Missing**	2	3.38	4	6.25
Subarachnoid hemorrhage	Yes	29	49.1	35	54.6
	No	28	47.4	25	39.0
	Missing**	2	3.38	4	6.25
Subdural hematoma	Yes	21	35.5	32	50.0
	No	36	61.0	28	43.7
	Missing**	2	3.38	4	6.25
Epidural hematoma	Yes	17	28.8	12	18.7
	No	40	67.7	48	75
	Missing**	2	3.38	4	6.25
Midline shift	Yes	12	20.3	14	21.8
	No	45	76.2	45	70.3
	Missing**	2	3.38	5	7.81
	Diffuse cerebral edema	Yes	0	0.0	1	1.56
No		57	96.6	59	92.1
Missing**		2	3.38	4	6.25

CT, computerized tomography. ^*^Lesion refers to intraparenchymal hemorrhage and/or contusion. ^**^Missing refers to CT images not found for review.

Treatment compliance was assessed based on both the patient’s electronic record and the drug accountability provided by the pharmacy and nurse team. In general, patients received mean (SD) 30.95 (11.59) doses of placebo initiating 12.11 (9.99) h after TBI or mean (SD) 25.79 (14.15) doses of biperiden initiating 10.65 (5.84) h after TBI, which is far below the total of 40 planned doses. More specifically, only 31/56 (55.3%) participants received at least 30 of the total of 40 doses (75%) of biperiden. Importantly, it must be considered that patients evolving to death before completing the intervention contributed to poor adherence numbers. Even so, biperiden was well tolerated for patients in the context of acute TBI. Constipation (43.9%; placebo *n* = 28, biperiden *n* = 26) and agitation (21.1%; placebo *n* = 10, biperiden *n* = 16) were the most common adverse events observed during the intervention period. Nevertheless, both events were primarily associated with prolonged bed rest and reduced levels of sedation in intensive care patients, respectively for constipation and agitation.

Death cases were mainly attributed to the TBI itself (placebo *n* = 9, biperiden *n* = 12); in other cases, deaths were attributed to injuries in other parts of the body (*n* = 1 placebo), comorbidities (*n* = 1 biperiden), sepsis (*n* = 1 placebo, 583 days; *n* = 1 biperiden, 82 days), malnutrition associated with major depressive disorder (*n* = 1 biperiden, 130 days) and neurologic shock (*n* = 1 biperiden, 214 days). One patient (placebo) had a stroke after 32 months and was not included in the mortality analysis. Cox regression models, both unadjusted and adjusted for GCSoS, showed that there is a lack of evidence regarding group differences for survival (Table 3).

**Table 3:** Results of the Cox regression models.

Outcome	Model	Coefficient	SE	*p*-value	HR	95% CI	
PTE	Unadjusted	0.97	0.70	0.170	2.63	0.65	10,57
	Adjusted	0.99	0.75	0.190	2.70	0.61	11,97
	Adjusted SHR	0.86	0.71	0.229	2.37	0.58	9.61
Survival	Unadjusted	0.45	0.39	0.248	1.57	0.73	3,38
	Adjusted	0.25	0.40	0.529	1.29	0.58	2,85

PTE, post-traumatic epilepsy; SHR, subdistribution hazard ratio derived from the competing risk regression.

For the primary outcome, 3 placebo patients and 6 patients in the biperiden group developed epilepsy during the two-year follow-up. An additional participant from the biperiden group presented alcohol withdrawal seizures 31 months after TBI, and therefore was not included on PTE analysis. Again, both Cox regression models, unadjusted and adjusted for GCSoS, showed lack of evidence regarding group differences for this outcome. The same was observed for the competing risk regression. Table 3 also shows the unadjusted and adjusted effects of the intervention for PTE outcome.

For the secondary outcome, the difference in the logs of expected counts of unprovoked seizures is expected to be 2.03 unit higher for biperiden compared to placebo (robust standard error = 0.573, *p*-value <0.001, 95% CI = 0.912 to 3.1597). After the adjustment of GCSoS, the results are close to what was previously reported, the difference in the logs of expected counts of unprovoked seizures, is expected to be 1.857 unit higher for biperiden compared to placebo (robust standard error = 0.762, *p*-value 0.015, 95% CI = 0.263 to 3.351).

## Discussion

This first randomized, double-blinded trial of treatment with biperiden versus placebo in patients with acute TBI shows that biperiden is generally safe and well-tolerated when used under the current administration regimen in critical care patients. For the primary outcome, incidence of PTE after TBI, results did not achieve significance when comparing both groups. Similar finding was achieved for survival analysis indicating lack of evidence.

For the secondary measurement, specifically late seizure frequency, this study indicated an increased count of unprovoked epileptic seizures in patients treated with biperiden during the two-year follow-up. In spite of baseline characteristics of groups being statistically similar, patients treated with biperiden tended to show more severe injuries as demonstrated by lower score at GCSoS than patients treated with placebo. Also, bilateral brain lesions were more frequent in the biperiden group. Both characteristics, severe and bilateral lesions, were already described as risk factors for PTE (22). Although statistical differences in seizure frequency were still found among groups even after adjusting for GCSoS in the present study, we speculate that group differences in these parameters might have contributed to difficulties in seizure control, as well as a possible lower ASM adherence following the first PTE seizure, which unfortunately was not controlled in the present study.

Balancing the different experimental groups concerning trauma severity or GCSoS score would have required a distinct experimental design increasing the overall complexity of conducting the trial, which we could not handle with the available resources. Inclusion criteria in the current study required not only GCS score between 3–12 but in addition TC scan with evidence of contusion or intraparenchymal hemorrhage. Additionally, efforts were made to ensure the treatment initiation time window, grounded on laboratory evidence, to be as soon as possible. Moreover, conducting a double-blind, placebo-controlled intervention in an emergency setting entail achieving a balance that is the least disturbing for the appropriate standard treatment that patients would typically receive, alongside the requisites of the clinical investigation. In conclusion to this aspect, while acknowledging the potential benefits of further defining patient allocation based on lesion aspects, doing so might introduce a significant burden to the study and could considerably compromise its feasibility.

Despite the variability in the incidence of PTE across different studies (23), considering collective findings, an occurrence rate of PTE in the current study would have been anticipated to be around 13–37% (20, 22, 24, 25–29), but the total incidence, 8.03% (9/112), was lower than expected, especially considering the 5.35% (3/56) observed cases for the placebo group. Still, this incidence is higher than the 2.7% diagnosis of PTE described in recent studies (5, 30). Considering this variability, the incidence rate should be carefully rethought to achieve validated sample size calculation through further investigation in the field.

Whereas most studies that present data on the incidence of epilepsy development after TBI typically have an inclusion window of 24 h for intervention, in this study, the interval was restricted to patients admitted in the initial 12 h after injury. There is no reported evidence on any potential influence of the delay for medical assistance after a lesional event and the subsequent development of PTE. Yet it is conceivable that a shorter timeframe for medical intervention might increase the likelihood of a favorable outcome for most medical conditions. In pre-clinical studies, anticholinergic treatment is known to potentially modify the epileptogenic process (10, 31, 32). Specifically, biperiden suppresses spontaneous seizures in animal models of epilepsy. In addition, treatment with biperiden can delay the latency and decrease the incidence and intensity of spontaneous seizures (31). Seconding the hypothesis, a recent paper suggested that scopolamine exerts antiepileptogenic/disease-modifying activity in the lithium-pilocarpine rat model, possibly involving increased remission of epilepsy as a new mechanism of disease-modification (33). Because of the robust results of biperiden and other anticholinergic drugs over suppressing the epileptogenic process in animal models, the inconclusive or even not beneficial use of biperiden in the current trial could be derived from the limitations of this study, as further discussed.

Based on experimental data from animal models it was hypothesized that among the critical factors for an effectiveness for preventing PTE are the time-window for starting treatment after injury, treatment duration and drug dosage. All of those parameters have only been tested in rats and the inferred parameters employed here may need adjustments. Moreover, difficulties were encountered, especially in the beginning of the study, to initiate the intervention within the scheduled 12 h after TCE, since many patients from primary health centers arrived at the tertiary referral hospital at the limit or suppressing this restricted time-window. In addition, ensuring intervention during the first 24 h, especially while patients were unstable or undergoing surgery, as well as maintaining intervention throughout 10 consecutive days, were also challenging, given the diversity of health professionals involved in patient care (whom many times were polytraumatized), and different hospitalization units inside the hospital (which is the largest hospital complex in Latin America). Together these factors contributed to protocol deviations.

Other major issues for this study was related to patient recruitment, which can be explained considering the characteristics of a teaching hospital, with high turnover of neurosurgery medical residents and the complex nature of the trial (unstable patients in the emergency room, need to quickly check eligibility criteria of possible participant, quickly obtain a cranial CT and correctly analyze lesion images, perform the randomization and administration of the first dose within 12 h of trauma), which increased the occurrence of screening failure.

Also as a limitation, while randomization was performed using random numbers generated by statistical software, the allocation of the medication occurred by sequential inclusion of participants, which may have induced potential bias, especially considering that it was not adjusted by trauma severity. Second, operational challenges had to be addressed, mainly related to the low-income characteristic of the population attended by the hospital, which added difficulty to contact and transport participants to follow-up visits, reduced operational research time, and finally the SARS-CoV-2 pandemic involving social distance policies, which limited our ability to adhere strictly to the designed follow-up schedule.

Indeed, the COVID-19 pandemic significantly impacted the study. Initially, patient enrollment was compelled to halt due to the stringent measures imposed as a response to the pandemic. Various countries adopted diverse strategies to manage this severe public health crisis, and there was no clear indication of the duration for which measures like social distancing and remote work would remain in effect. Furthermore, acknowledgement was made of the potential presence of additional variables introduced by the pandemic, which could hinder the amalgamation of data collected before and after the pandemic. Consequently, the determination to conclude the study was made. This decision not only facilitates a more thorough and comprehensive analysis of the results but also paves the way for potential adjustments in preparation for a new clinical trial.

As aforementioned, we did not find clinical evidence for the primary endpoint, the number of patients developing PTE. Unexpectedly, the use of biperiden in patients after TBI might worsen the frequency of seizures in those patients that develop PTE. Supported by these uncertain findings, planning for an experimental design in a new multicenter trial will be refined to incorporate a larger sample size in an efficient time schedule; more restricted inclusion criteria limited only for patients with moderate and severe lesions as per GCS on admission; blind randomization by each center *in loco*, and for each patient (instead of sequentially); electronic case report forms using the RedCap system for data collection, reinforcement of protocol training and of structured research teams. These changes will help to constrain variability and increase study quality.

Despite decades of experimental investigation into the plastic changes that ensue after lesion events in the brain, there has been little progress using this concept into clinical testing. EpiBioS4Rx, a large collaborative effort currently being carried out is expected to yield one or more candidate antiepileptogenic treatments, as well as biomarker information, resources, expertise, and patient populations sufficient to carry out an economically feasible, full-scale clinical trial of at least one antiepileptogenic intervention (16). The results of our study directly anticipate some of the issues that should be considered when designing such trials, including those related with the time window after the precipitating injury.

## Conclusion

There was insufficient evidence regarding the effect of biperiden in preventing post-traumatic epilepsy after TBI. The combined effect of variables known to have an impact on the likelihood of developing late post-traumatic seizures and its unbalanced frequency in the different groups is an aspect to be considered and underpins the need for larger studies.

## Transparency, rigor, and reproducibility summary

The study design and analysis plan were preregistered on January 13, 2010 at https://www.clinicaltrials.gov/study/NCT01048138, under NCT01048138. Prespecified sample size was 57 per group, yielding statistical power of 80% for detection of an effect size of 5% for the primary outcome measure. All subjects were assigned to biperiden or placebo using a random number generator, yielding groups that did not differ in baseline characteristics. 123 subjects were engaged and primary outcomes were assessed in 112 subjects after 27 deaths and 14 incomplete assessments. All primary outcomes were assessed by investigators blinded to group assignment and could guess the group assignment with accuracy no greater than chance. Biperiden required to perform the interventions are widely available from Cristália (Brazil). Key inclusion criteria were assessed by investigators with professional qualifications (medical residents). Clinical outcomes were assessed by investigators with extensive professional qualifications (neurologists and neurophysiologists). Statistical analysis was performed by researcher with extensive experience in statistical analysis for clinical trials. Ongoing replication studies have been preregistered at https://www.clinicaltrials.gov/study/NCT04945213. De-identified data from this study are not available in a public archive. De-identified data from this study will be made available (as allowable according to institutional IRB standards) by emailing the corresponding author.

## Data availability statement

The raw data supporting the conclusions of this article will be made available by the authors, without undue reservation.

## Ethics statement

The studies involving humans were approved by Research Ethics Committee of Hospital das Clínicas da Faculdade de Medicina da Universidade de São Paulo (HC-FMUSP; CAAE n 08533513.6.2002.0068) and Universidade Federal de São Paulo (UNIFESP; CAAE n 08533513.6.1001.5505). The studies were conducted in accordance with the local legislation and institutional requirements. The participants provided their written informed consent to participate in this study.

## Author contributions

MF: Writing – review & editing, Writing – original draft, Validation, Supervision, Project administration, Methodology, Investigation, Formal analysis, Data curation, Conceptualization. EG: Writing – review & editing, Writing – original draft, Validation, Supervision, Methodology, Investigation, Data curation, Conceptualization. MM: Writing – review & editing, Methodology, Investigation, Data curation. RV: Writing – review & editing, Methodology, Investigation, Data curation. JS: Writing – review & editing, Methodology, Investigation, Data curation. GS: Writing – review & editing, Methodology, Investigation, Data curation. NL: Writing – review & editing, Methodology, Investigation, Data curation. CB: Writing – review & editing, Investigation, Data curation. JA: Writing – review & editing, Methodology, Investigation. MS: Writing – review & editing, Methodology. CL: Writing – review & editing, Data curation. MN: Project administration, Methodology, Writing – review & editing. WP: Writing – review & editing, Methodology. AA: Writing – review & editing, Supervision, Methodology. MT: Writing – review & editing, Investigation. LM: Writing – review & editing, Writing – original draft, Visualization, Validation, Supervision, Resources, Project administration, Methodology, Investigation, Funding acquisition, Conceptualization.
